# Molecular characterization and differential expression suggested diverse functions of P-type II Ca^2+^ATPases in *Triticum aestivum* L

**DOI:** 10.1186/s12864-018-4792-9

**Published:** 2018-05-23

**Authors:** Mehak Taneja, Santosh Kumar Upadhyay

**Affiliations:** Department of Botany, Panjab University, Chandigarh, 160014 India

**Keywords:** *Triticum aestivum*, Ca^2+^ATPases, ACAs, ECAs, Heat, Drought, Salt, Biotic stress

## Abstract

**Background:**

Plant P-type II Ca^2+^ATPases are formed by two distinct groups of proteins (ACAs and ECAs) that perform pumping of Ca^2+^ outside the cytoplasm during homeostasis, and play vital functions during development and stress management. In the present study, we have performed identification and characterisation of *P-type II Ca*^*2+*^*ATPase* gene family in an important crop plant *Triticum aestivum*.

**Results:**

Herein, a total of 33 TaACA and 9 TaECA proteins were identified from the various chromosomes and sub-genomes of *Triticum aestivum*. Phylogenetic analysis revealed clustering of the homoeologous TaACA and TaECA proteins into 11 and 3 distinct groups that exhibited high sequence homology and comparable structural organization as well. Both TaACA and TaECA group proteins consisted of eight to ten transmembrane regions, and their respective domains and motifs. Prediction of sub-cellular localization was found variable for most of the proteins; moreover, it was consistent with the evolutionarily related proteins from rice and Arabidopsis in certain cases. The occurrence of assorted sets of *cis*-regulatory elements indicated their diverse functions. The differential expression of various *TaACA* and *TaECA* genes during developmental stages suggested their roles in growth and development. The modulated expression during heat, drought, salt and biotic stresses along with the occurrence of various stress specific *cis*-regulatory elements suggested their association with stress response. Interaction of these genes with numerous development and stress related genes indicated their decisive role in various biological processes and signaling.

**Conclusion:**

*T. aestivum* genome consisted of a maximum of 42 *P-type II Ca*^*2+*^*ATPase* genes, derived from each A, B and D sub-genome. These genes may play diverse functions during plant growth and development. They may also be involved in signalling during abiotic and biotic stresses. The present study provides a comprehensive insight into the role of *P-type II Ca*^*2+*^*ATPase* genes in *T. aestivum*. However, the specific function of each gene needs to be established, which could be utilized in future crop improvement programs.

**Electronic supplementary material:**

The online version of this article (10.1186/s12864-018-4792-9) contains supplementary material, which is available to authorized users.

## Background

Calcium ion (Ca^2+^) plays numerous crucial functions during growth and development of plants due to its role as a secondary messenger and as an essential element [1]. It is also involved in stress response to various biotic and abiotic stimuli [2]. Ca^2+^ concentration usually increases during stress which is sensed by sensor proteins or calcium-binding proteins (CBPs) that start signalling cascade for adaptation [3]. The homeostasis of Ca^2+^ inside the cell is regulated by the functioning of three major transporters- Ca^2+^ permeable channels, Ca^2+^/cation antiporters (CaCAs) and Ca^2+^-ATPases [4].

Plant Ca^2+^ATPases belong to the P-type ATPases superfamily characterized by (1) the formation of phospho-aspartate enzyme intermediate during the course of a reaction, (2) occurrence of numerous conserved motifs and (3) vanadate inhibition [5, 6]. They perform pumping of Ca^2+^ outside the cytoplasm during homeostasis [7]. Structurally, these enzymes consist of eight to twelve transmembrane (TM) helices and five well-defined domains, three of them (A, N, and P) are cytoplasmic while two (T and S) are situated in the membrane [8]. Further, they consist of nine characteristic motifs which perform specific functions. All P-type ATPases show a common mechanism for ion transport. They usually undergo conformational changes from E1 to E2 and vice versa by phosphorylation and dephosphorylation processes during metal ion transport [9].

Plant Ca^2+^ATPases are phylogenetically classified into two distinct subgroups, type IIA (P2A) and type IIB (P2B), which are also known as ECAs (endoplasmic reticulum calcium ATPases) and ACAs (auto-inhibited calcium ATPases), respectively. They differ with respect to the absence or presence of N-terminal autoinhibitory domain, which activates the Ca^2+^ pump upon binding to calmodulin [5, 10]. Both type IIA and IIB Ca^2+^ATPases may be located at either endomembrane system or plasma membrane (PM) in plants. However, they are exclusively present in endoplasmic reticulum (ER) and PM in animals, respectively [11].

Several studies have been carried out to characterize the selected Ca^2+^ATPases in various plant species, which suggested their involvement in a wide array of phenomenon such as vegetative and reproductive tissue development, stomatal movement, hormonal signalling and fertilisation, and in various immunological, biotic and abiotic stress responses [10, 12]. Despite of their vital functions, the genome-wide identification and characterizations have been performed in limited plant species like Arabidopsis, rice, and sorghum [4, 10, 13].

*T. aestivum* (bread wheat) is a staple food crop consumed by about one-third of the total world population. Therefore, there is a grave need to perform the characterization of its every aspect at the genome scale. A comprehensive analysis of numerous gene families has not been carried out earlier due to the lack of its complete genomic information, allohybridized genome and intricate tools for functional genomics. Introduction of high-throughput sequencing technologies and evolved computational approaches in recent years ease the genome-scale characterization of various gene families. Recently, the genome of *T. aestivum* has also been decoded by various groups [14, 15], and numerous high throughput RNA sequence data are generated from various developmental stages and stress treatments [16–19], which facilitated characterization of many gene families [20, 21].

Earlier, we performed characterization of Ca^2+^/cation antiporters (CaCAs) protein superfamily in *T. aestivum* and its A and D sub-genome progenitors [22], which is one of the three major transporters facilitating Ca^2+^ homeostasis in plants. In the current study, we aimed genome-wide identification, characterization, expression profiling, and co-expression analyses of type II Ca^2+^ATPases in various tissue developmental stages and in the presence of numerous stresses in *T. aestivum*. During the compilation of our findings, we found that Aslam et al., (2017) [23] have also identified Ca^2+^ATPases in *T. aestivum*. However, they had not performed inclusive characterization of complete gene family. Further, the expression study was solely carried out under calcium stress. We identified additional type IIB Ca^2+^ATPase proteins and performed detailed gene and protein structural characterization. We also analyzed the occurrence of various *cis*-regulatory elements and documented the genes specifically involved in various tissue developmental processes and biotic (against fungal pathogens) and abiotic (heat, drought, and salt) stress responses.

## Results and discussion

### Identification and classification of type II Ca^2+^ATPase proteins in *T. aestivum*

We identified a total of 42 proteins as putative type II Ca^2+^ATPases in the *T. aestivum* genome by an extensive BLAST search, which were classified into 33 TaACA and 9 TaECA proteins based on the presence or absence of N-terminal autoinhibitory (CaATP_NAI) domain, respectively (Additional file 1: Table S1). However, CaATP_NAI domain was absent in TaACA2 and TaACA6 despite of their high homology with other ACA proteins. In the earlier studies, a total of 15 type II Ca^2+^ATPase proteins are reported in Arabidopsis and rice, and 14 in sorghum [4, 10, 11, 13]. The results indicated that allohexaploid genome of *T. aestivum* comprised of about thrice the number of type II Ca^2+^ATPase proteins as compared to the other diploid plant species. Our results were consistent with the earlier reports of the presence of higher number of genes in the other gene families of *T. aestivum* including another family of calcium transporters i.e. Ca^2+^/Cation antiporters [20–22]. Further, two (LOC_Os02g08010 and LOC_Os08g40530) out of 12 rice ACA proteins also lacked CaATP_NAI domain [10] as observed in case of TaACA2 and TaACA6. Aslam et al., (2017) [23] identified only 27 TaACA proteins; they had not reported *TaACA4* genes. Furthermore, we identified a total of 14 (11 TuACAs and 3 TuECAs) and 15 (12 AeACAs and 3 AeECAs) type II Ca^2+^ATPase proteins in the *T. urartu* and *Ae. tauschii* genomes (Additional file 1: Table S1) that constitute the A and D sub-genomes of *T. aestivum*, respectively [24, 25].

### Sub-genomic localization, homoeologous grouping, and chromosomal distribution

**Fig. 1:**
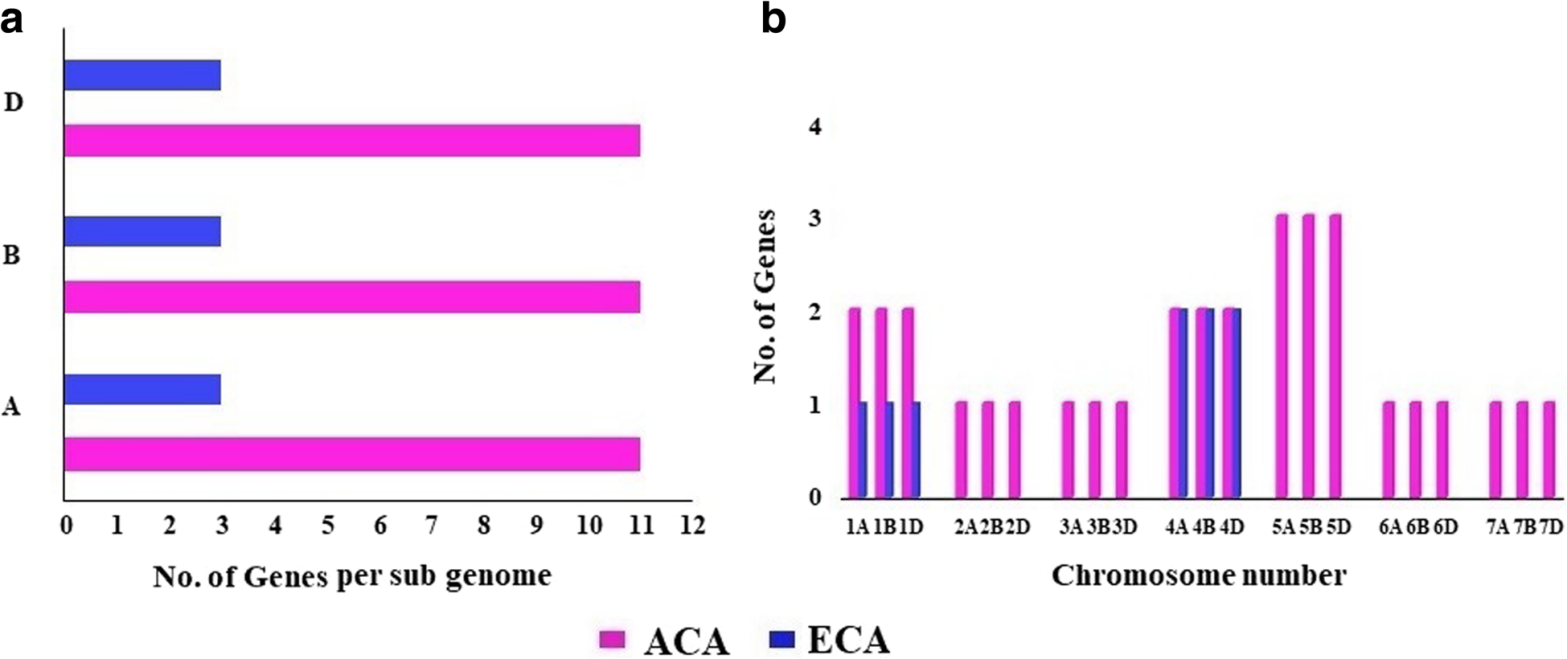
Distribution of the *TaACA* and *TaECA* genes on A, B and D subgenomes and chromosomes. Figure shows the frequency of both *TaACA* and *TaECA* genes on various chromosomes (**a**) and on A, B and D sub-genomes (**b**). Each sub-genome contributed equally towards the genomic composition of type II *Ca*^*2+*^*ATPases* in the *T. aestivum* genome

Genome-wide analyses revealed that all the three sub-genomes contributed uniformly towards the composition of type II Ca^2+^ATPase proteins in *T. aestivum* genome (Fig. 1a). Eleven *TaACA* and three *TaECA* genes were derived from each of the A, B and D sub-genome. Due to an allohexaploid nature of *T. aestivum* genome, the majority of genes are usually derived from the homoeologous chromosomes of each sub-genome, and thus shares very high sequence homology [20–22]. The results revealed a total of 11 and 3 distinct clusters of homoeologous *TaACA* and *TaECA* genes, respectively (Additional file 2: Table S2). The number of predicted homoeologous groups was comparable to the total number of genes reported in diploid plants like Arabidopsis and rice [10, 11].

In total 32 *TaACA* genes were mapped on all the 7 chromosomes, which forms the monoploid number of bread wheat (Fig. 1b). Location for one gene could not be determined on any chromosome and was named *TaACA11-A* based on the chromosomal location of its homoeologous IWGSC gene id (Additional file 1: Table S1). A maximum of three genes (*TaACA7*, *TaACA8*, and *TaACA9*) were located on the chromosome group 5, whereas two genes each on chromosome group 1 (*TaACA1*, *TaACA2*) and 4 (*TaACA5* and *TaACA6*). In case of *ECA*, two and one genes were located on chromosome group 4 and 1, respectively (Fig. 1b). In the other plant species, *type II Ca*^*2+*^*ATPases* are variably distributed on different chromosomes. For instance, a maximum number of genes are located on chromosome 3 in rice, whereas chromosomes 6, 7 and 9 are devoid of *type II Ca*^*2+*^*ATPases* [10]. Interestingly, these chromosomes lack all the Ca^2+^ transporters including exchangers, ATPases, and channels [13]. In sorghum, *type II Ca*^*2+*^*ATPases* are majorly located on chromosome 1, 3 and 4 [13]. However, in Arabidopsis, 3 out of 4 *type IIA Ca*^*2+*^*ATPase* genes are located on chromosome 1, and most of the *type IIB Ca*^*2+*^*ATPases* are positioned on chromosome 3 [10].

### Phylogenetic analysis and gene duplication

**Fig. 2:**
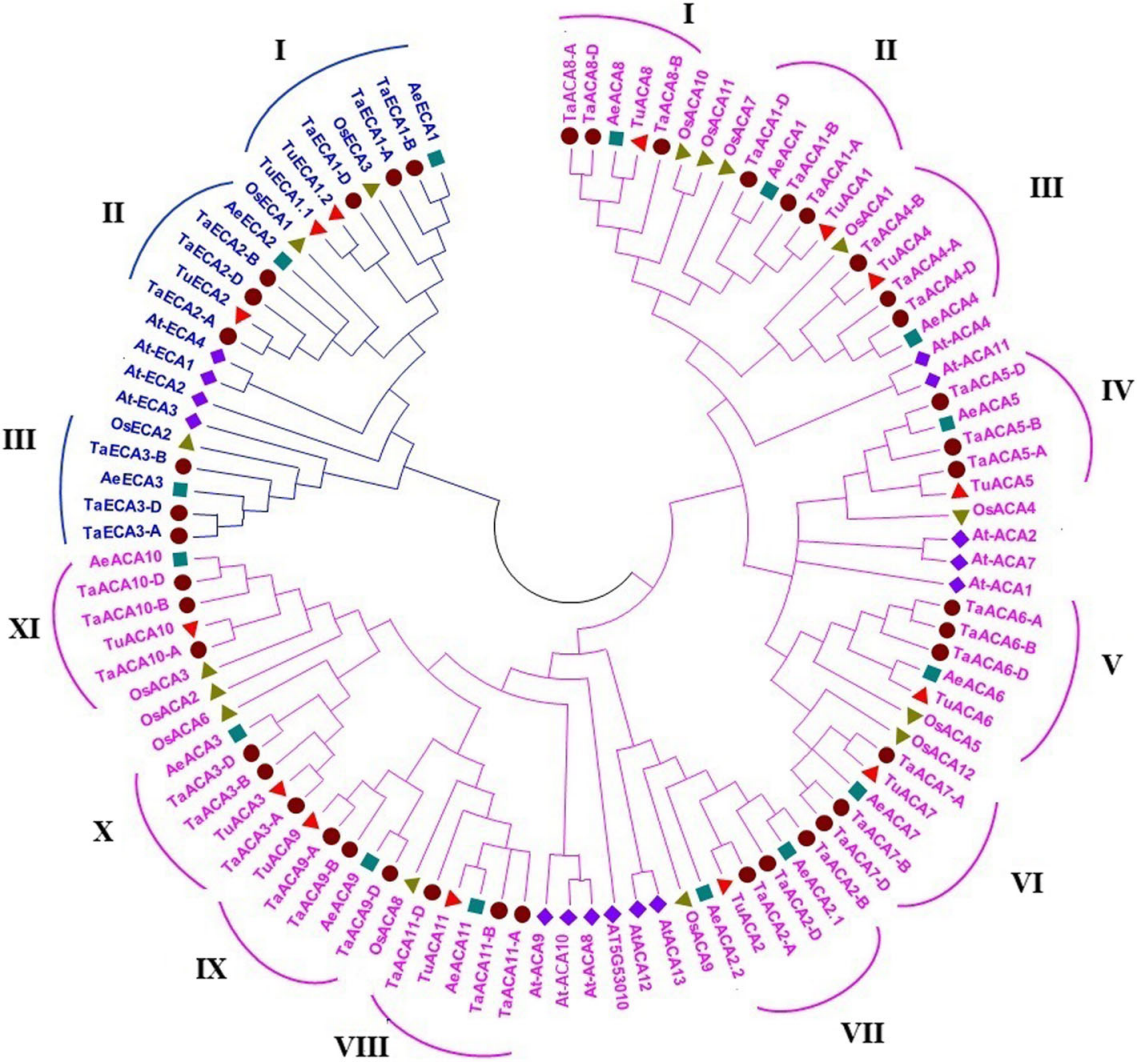
Phylogenetic analysis of P-type II Ca^2+^ATPase proteins. The evolutionary relatedness between type IIA and type IIB Ca^2+^ ATPase proteins of *T. aestivum, T. urartu*, *Ae. tauschii*, Arabidopsis and rice were analysed using the full length amino acid sequences. Figure shows clustering of ACA and ECA proteins from different plant species, that are represented by pink and blue colours, respectively. The ACA and ECA subgroups are further divided into 11 and three distinct groups, respectively, which includes the homoeologous genes in both the subgroups

A phylogenetic tree was constructed using the full length sequences of P-type II Ca^2+^ATPase proteins from *T. aestivum, T. urartu*, *Ae. tauschii*, Arabidopsis and rice. Both ACA and ECA proteins formed two distinct groups that could be further divided into clades and subclades (Fig. 2). The homoeologous TaACA and TaECA proteins of *T. aestivum* were tightly clustered together due to a high degree of homology between them. Monocot ACAs and ECAs formed 11 and 3 distinct clades as per the homoeologous grouping of TaACA and TaECA proteins, respectively. However, OsACA2 and OsACA3 were grouped together in clade XI and Arabidopsis ACA and ECA proteins were found assorted in its own respective groups. The proteins identified from *T. urartu* and *Ae. tauschii* were tightly grouped with their counterparts derived from the A and D sub-genomes of *T. aestivum*, respectively. Similar clustering has also been reported in case of other gene families of *T. aestivum, T. urartu* and *Ae. tauschii* [20–22, 26].

*Ae. tauschii* genome consisted of 12 AeACA proteins as compared to the 11 TaACA proteins found in the D sub-genome of *T. aestivum*. We observed that two AeACA proteins i.e. AeACA2.1 and AeACA2.2 exhibited a very high degree of similarity with each other, and tightly clustered with TaACA2 group proteins in clade VII. The results suggested that either *T. aestivum* lost one TaACA2 group protein from its D sub-genome or *Ae. tauschii* gained an additional AeACA2.2 protein by gene duplication after hybridization event. Similarly in case of ECA, *T. aestivum* comprised three TaECA homoeologous group proteins which were distributed in each clade. But two (TuECA1.1 and TuECA1.2) out of three ECA proteins of *T. urartu* displayed tight clustering with TaECA group 1 proteins. Further, no TuECA protein was found in the clade III. The results suggested that the genome of *T. aestivum* must have undergone loss of one *TaECA1* group gene from the A sub-genome and gained an additional *TaECA3* gene in the same sub-genome by an event of gene duplication. The occurrence of *TaECA2-A* and *TaECA3-A* on the two arms (S and L) of chromosome 4A (Additional file 3: Table S3), pericentromeric inversion of chromosome 4AS-4AL [27] and numerous incidence of translocation in *T. aestivum* genome [28, 29] further strengthen the prevalence of gene duplication in *TaECA* genes.

### Structural characterization

**Fig. 3:**
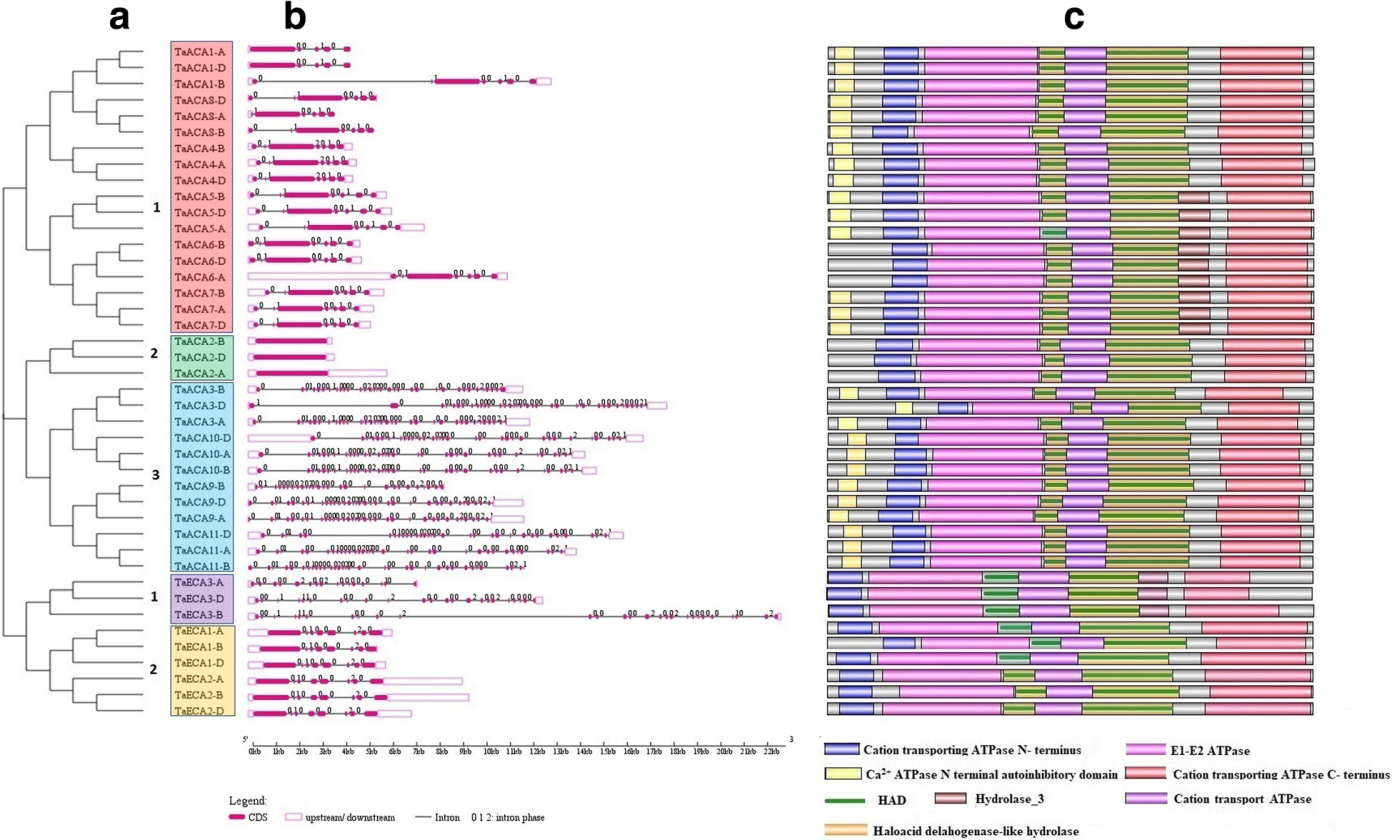
Structural analysis of genes and proteins. Phylogenetic relationship of TaACAs and TaECAs (**a**), Intron/exon configurations of both *TaACA* and *TaECA* genes (**b**), and domain organisation of TaACA and TaECA proteins (**c**). Exons and introns are shown as magenta colour boxes and thin lines, respectively. UTRs are shown with pink outlined boxes. 0 = intron phase 0; 1 = intron phase 1; 2 = intron phase 2; 3 = intron phase 3. The identified domains were represented by different colour boxes. *TaACAs* and *TaECAs* were clustered into three and two distinct groups based on exon/intron organization, respectively. All the major domains are present in each TaACA and TaECA group of protein

The *type II Ca*^*2+*^*ATPase* gene family in *T. aestivum* was analyzed for several characteristic features. The *TaACA* and *TaECA* genes formed three and two distinct groups on the basis of exon/intron organization, respectively (Fig. 3a). Genes in each group were tightly clustered and exhibited close evolutionary relationship among them and with the genes of the other groups, as well. The majority of *TaACA* genes in group 1 and group 3 consisted of seven and 34 exons, respectively. However, group 2 *TaACA* genes were intronless. Similarly, each *TaECA* gene of group 1 possessed 8 exons, while group 2 showed an assorted number of exons. Most of the homoeologous genes consisted of similar exon/intron distribution. Similarly, the majority of Arabidopsis and rice *ACA* genes are also having seven exons, while a maximum of 34 exons are present in *OsACA6*, and 35 exons in each *At-ACA8* and *At-ACA10* [10].

Intron phase analysis in *TaACA* genes revealed the occurrence of 78.6, 12.6, 7.4 and 1.3% introns in phases 0, 1, 2 and 3, respectively. In *TaECA* genes, there were 73% introns in the 0 phase, while 13.4% introns were in each phase 1and 2 (Fig. 3b). Our finding was consistent with the earlier reports that the majority of eukaryotic genes including the calcium transport-related genes possess conserved phase 0 introns, which are least affected by selection pressure in the due course of evolution [22].

The length of TaACA proteins varied from a minimum of 1014 amino acid residues [AAs] in TaACA8-B to a maximum of 1228 AAs in TaACA3-D with an average length of ~1056 AAs (Table 1; Additional file 3: Table S3). In case of Arabidopsis and rice, the longest and shortest ACA proteins are AtACA9 (1086 AAs) and AtACA2 (1014 AAs), and OsACA6 (1089 AAs) and OsACA3 (326 AAs), respectively. Among ECAs, TaECA1-B exhibited a maximum length of 1132 AAs and, TaECA3-A and D had a minimum length of 939 AAs each, while the average residue number was 1040 AAs. The maximum length of ECA proteins in rice and Arabidopsis are 1218 AAs (OsECA2) and 1061 AAs (AtECA1 and AtECA4), whereas the shortest proteins are OsECA3 (374 AAs) and AtECA3 (998 AAs), respectively. The molecular weight (MW) of TaACA proteins varied from ~133 kDa to ~110 kDa of TaACA3-D and TaACA2-B, respectively. In case of TaECAs, it ranged from a maximum of ~122 kDa in TaECA1-B to a minimum of ~102 kDa in TaECA3-D. Average MW for both plants and mammalian type II Ca^2+^ATPases are earlier reported to be in the same range [5]. The isoelectric point (pI) of TaACA and TaECA proteins ranged from 4.6 to 8 and 4.9 to 6.4, respectively (Table 1). Positive GRAVY value of each Ca^2+^ATPase protein suggested their hydrophobic nature except TaACA11 group proteins, which exhibited negative GRAVY scores.

**Table 1:** General characteristic features of P-type II Ca^2+^ATPase proteins of *T. aestivum*

		TaACAs	TaECAs
Protein length (AAs)	Maximum	1228	1132
	Minimum	1014	939
	Average	1055.7	1040
MW (kDA)	Maximum	133.6	122.8
	Minimum	109.9	102.4
	Average	114.9	113.5
pI	Maximum	8.2	6.4
	Minimum	4.7	4.9
	Average	6.5	5.5
Loop I size	Maximum	137	195
	Minimum	136	149
	Average	136	155
Loop II size	Maximum	391	434
	Minimum	381	429
	Average	386	432

The occurrence of TM regions was predicted using various tools. Since the different tools employ different algorithms and parameters for their computation; an average value was calculated. Eight to ten TM regions were predicted in the majority of TaACA and TaECA proteins (Additional file 4: Table S4; Additional file 5: Figure S1). The biochemical studies have revealed the presence of ten TM helices in mammalian type IIA Ca^2+^ATPases [30]. Moreover, the incidence of eight TM helices is also reported in plant Ca^2+^ATPases [31]. The TMs usually form two membrane-embedded distinct domains, T (Transport) and S (class specific transport domain). The T-domain consists of an ion binding site(s) and it is formed by the first six TM helices. The remaining four TM helices constitute the S-domain that structurally supports the T-domain and provides ion-coordinating side chains for additional ion-binding sites in both Ca^2+^ and Na^+^/K^+^-ATPases [8].

Furthermore, two distinct cytoplasmic loops I and II, connecting TM2 and TM3 and TM4 and TM5, were detected in each type II Ca^2+^ATPase protein of *T. aestivum*, respectively. Most of the TaACA proteins comprised 136 AAs long loop I, while loop II varied from 381 to 391 AAs. In case of TaECAs, the size of loop I and loop II ranged from 149 to 195 and 429 to 434 AAs, respectively (Table 1; Additional file 3: Table S3). Loop I together with a linker sequence from TM1 is known to harbour the A (Actuator) domain, that is one of the three cytoplasmic domains present in P-type ATPases. However, the second and larger cytoplasmic loop referred as loop II possesses the remaining two cytoplasmic domains- N (nucleotide binding) and P (Phosphorylation). These cytoplasmic domains function together to perform ATP hydrolysis that results in the transport and counter transport of ions through the membrane [6, 32].

### Domains and motifs analyses

Domain analysis of any gene family plays a crucial role in determining its characteristic function. Each TaACA protein comprised of all the five major domains (CaATP_NAI, N-terminus cations transporter/ATPase domain, E1-E2 ATPase domain, haloacid dehalogenase-like hydrolase and C-terminus cations transporter/ATPase), except TaACA2 and TaACA6. The TaECA proteins also consisted of these domains, except CaATP_NAI (Fig. 3c; Additional file 6: Table S5). The CaATP_NAI possesses an autoinhibitory region along with a calmodulin (CaM) binding site and a serine residue that blocks activation of CaM. The pump gets activated when CaM binds the autoinhibitory region while its phosphorylation by CDPK (Ca^2+^ − dependent protein kinases) inhibits its activity [33]. Additionally, a cation transport ATPase domain was also identified in each type II Ca^2+^ATPase of *T. aestivum*. It is known to occur in cation transport ATPases, including phospholipid-transporting ATPases, calcium-transporting ATPases, and sodium-potassium ATPases [31]. A hydrolase_3 domain was also found in the TaACA5, TaACA6, TaACA7 and TaECA3 group proteins. It is present in the haloacid dehydrogenase superfamily (HAD) enzymes which perform cellular processes varying from amino acid biosynthesis to detoxification [34]. In case of *Ae. tauschii* and *T. urartu*, all the identified proteins consisted of their respective major domains, except AeACA2.2, AeACA4, TuACA3, TuACA4, AeECA1, AeECA2 and TuECA1.1, which lacked Cation_ATPase_N domain (Table S5). Further, type IIB Ca^2+^ATPase proteins like TuACA1, TuACA2, TuACA6 and TuACA8 among *T. urartu*, and AeACA1, AeACA2.1, AeACA2.2, AeACA6, AeACA8 and AeACA11 among *Ae. tauschii* were devoid of CaATP_NAI domain. However, they were grouped together with other ACA proteins because of its high sequence homology. Similar domain organization has also been reported in other plants type II Ca^2+^ATPase proteins [10, 13].

**Fig. 4:**
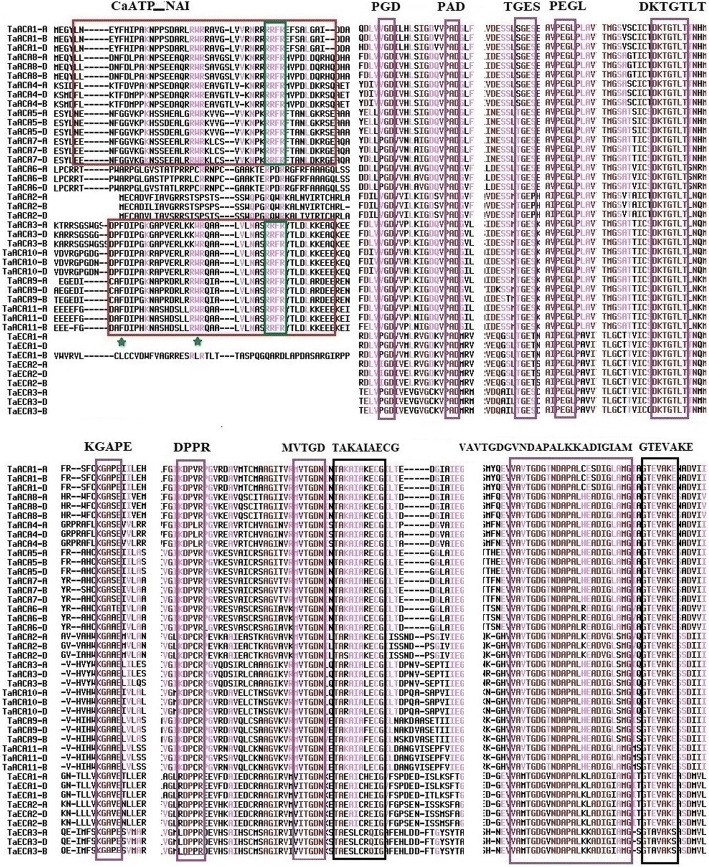
Multiple sequence alignment of P-type II Ca^2+^ATPases of *T. aestivum*. Figure shows alignment of amino acid sequences of type IIA and type IIB Ca^2+^ATPase proteins performed by multalin. The nine characteristic motifs (purple box); other reported conserved motifs (black box); the CaATP_NAI (calmodulin binding N-terminal auto inhibitory domain); the CaM binding sites (green colour) are highlighted in the figure. The presence of characteristic motifs and other conserved regions established their identity as P-type II Ca^2+^ATPases in *T. aestivum*

Multiple sequence alignment of 42 type II Ca^2+^ATPase proteins of *T. aestivum* was carried out to analyze the conserved motifs. All the nine common characteristic motifs of P-type ATPases [35] were detected in the present study (Fig. 4; Additional file 5: Figure S1). The first conserved motif (motif 1 or PGD) showed variation with respect to the first residue where P (Proline) is replaced by V (Valine) in some of the cases. However, the other two residues, G, and D were highly conserved among all the sequences. The other motifs which were found included ‘TGES’, ‘PEGL’, ‘DKTGTLT’, ‘KGAXE where X can be (P, S, V or F), ‘DPPR’, ‘MITGD’, ‘TAKAIAECG’, ‘GTEVAKE’ and the hinge motif. Similar motif distribution pattern and conservation are described in type II Ca^2+^ATPase proteins in various other organisms including plants [30]. The entirely conserved motifs among all the type II Ca^2+^ATPases of *T. aestivum* were motif 2 ‘PAD’, motif 4 ‘PEGL’, motif 5 ‘DKTGTLT’ and the last three residues ‘TGD’ of motif 8. The hinge motif ‘VAMTGDGANDAPALKKADIGIAM’, also called motif 9 was found conserved with the internal residues ‘TGDG’ and ‘NDAPAL’. The first three motifs were located in loop I, which are reported to provide stability to this region and facilitate conformational changes between E1 and E2 [7]. Fourth motif ‘PEGL’ is known to be associated with energy transduction [30], however, fifth motif ‘DKTGTLT’ holds the phosphorylation site at aspartate and lysine residues. Motif 6 ‘KGAPE’ is possibly involved in ATP binding [30]. Further, the aspartate and proline residues in DPPR (motif 7), and glycine and aspartate residues of TGD (motif 8) are known to play a vital role in phosphorylation. The ninth motif is possibly involved in facilitating ATP hydrolysis [30]. An additional sequence motif ‘RRFR’ and two completely conserved residues F and W of functional importance reported in CaATP_NAI of type IIB Ca^2+^ATPases, were also found conserved in the present study [33].

### Sub-cellular localization

According to earlier reports, the plant Ca^2+^ATPase proteins occur variably in multiple membrane systems including the PM, tonoplast, and inner membrane of plastid [5]. In contrast, type IIA and type IIB calcium pumps in animals are strictly localized in the endoplasmic reticulum and PM, respectively. In the present study, several tools were employed for the prediction of subcellular localization (Additional file 7: Table S6). The type IIA Ca^2+^ATPases were predicted to occur variably by the different tools used. CELLO and WoLF-PSort predicted all the TaECA proteins to be located in the PM whereas Prot Comp 9.0 predicted multiple locations. However, the tools like MultiLoc2 and BaCelLo predicted cytoplasm as preferred location for most of the ECA proteins.

In case of TaACAs, PM was the commonly predicted location by the majority of tools. Moreover, other cellular localizations such as chloroplast, mitochondria, endoplasmic reticulum and cytoplasm were also predicted by certain tools (Additional file 7: Table S6). Several reports established the cellular location of ACA and ECA proteins in rice and Arabidopsis. OsECA2 of rice reported to be localized in PM [11], was found evolutionary related to TaECA3 group proteins that shared similar localization. Similarly, AtACA8, AtACA9 and AtACA10 of Arabidopsis are known to be localized in PM [12] likewise its phylogenetically related TaACA9 and TaACA11 group proteins (Fig. 2).

### *Cis*-regulatory elements analysis

**Fig. 5:**
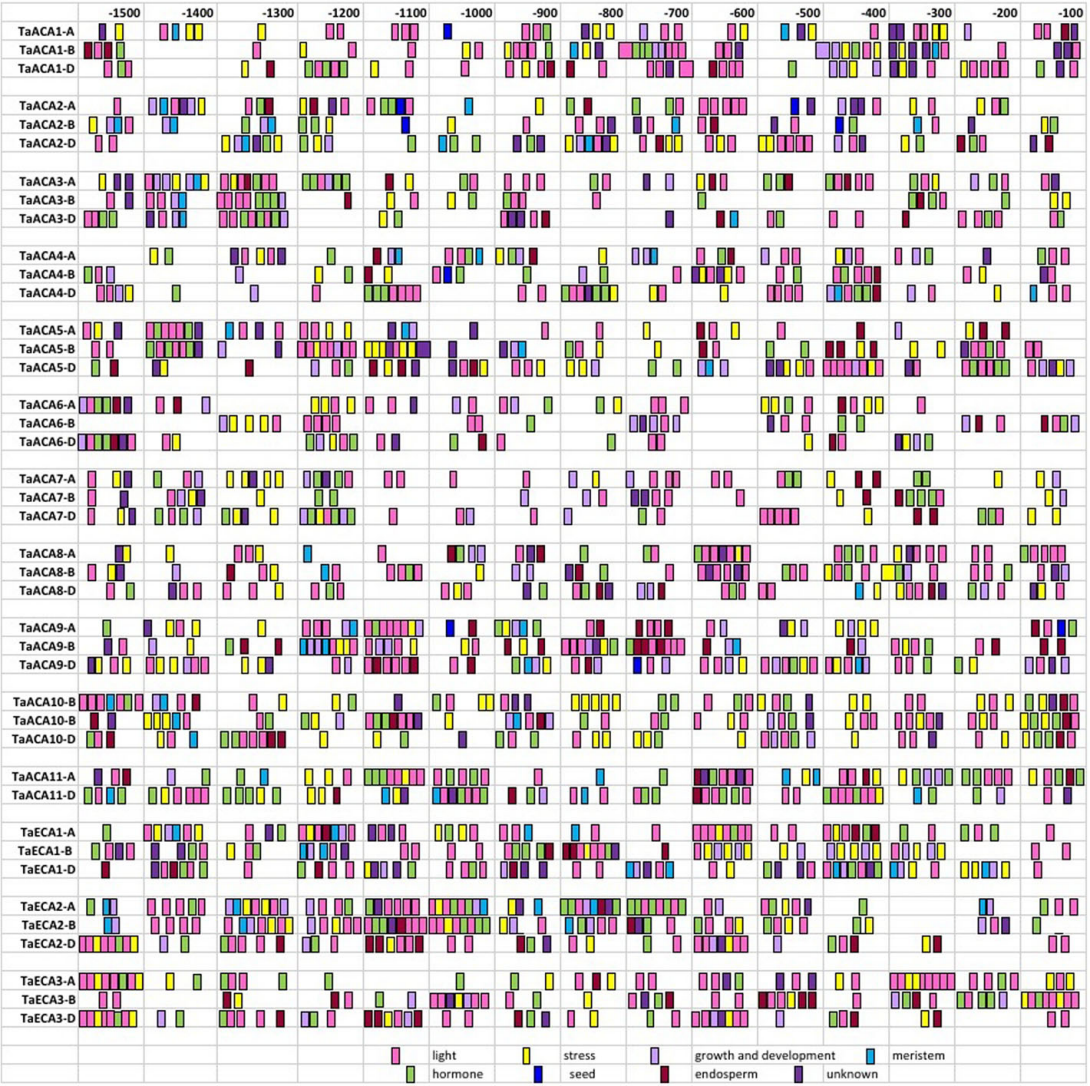
Distribution of cis regulatory elements in the upstream promoter regions of *TaACA* and *TaECA* genes. The elements were classified based on their function and indicated using different colours. Elements responsive to development, light, hormone and stress were indicated in mauve, pink, green and yellow, respectively. Elements for meristem, seed and endosperm specific expression were indicated by sky blue, dark blue and brown, respectively, while other elements with not any validated function were indicated by royal purple. The occurrence of different sets of *cis*-regulatory elements suggests diverse roles of type II Ca^2+^ATPases

Promoter region for all the genes was analysed for the occurrence of major cis-regulatory elements, except *TaACA11-B* for which no upstream region was found. The elements reported using the PLANTCARE database were broadly divided on the basis of their roles i.e. elements involved in growth and development, stress response, hormonal responsive, endosperm, seed, and meristem-specific and light responsive (Fig. 5). Further, a wide range of elements were also identified from PLACE database (Additional file 8: Table S7). Earlier studies on rice and Arabidopsis type II Ca^2+^ATPases documented the widespread occurrence of promoter elements belonging to the MYB and MYC family, supporting their role during various abiotic stress conditions [10, 13]. Similar results were obtained in the present study, where the commonly occurring elements that were identified in all the P-type II Ca^2+^ATPase genes of *T. aestivum* were GT1CONSENSUS, DOFCOREZM, GTGANTG10, GATABOX, ARR1AT, MYBCORE, POLLEN1LELAT52, ACGTATERD1, CACTFTPPCA1, WRKY71OS, and CURECORECR. The occurrence of these elements points towards their specific roles in growth and development, cell division, pollen tube growth and elongation [1]. Other abundantly occurring elements were ABRELATERD1, MYCATERD1, G-Box, LTRECORE, MYCATRD22, MYCCONSENSUAT, MYB1AT, MYB2, and HSELIKENTACIDICPR1 that function towards the amelioration of stress caused by external stimuli like salinity, pathogenic infections, drought, heat, and light [36]. Further, the presence of cis-element ABRERATCAL in the majority of the genes established their role in calcium responsiveness [10].

### Expression analyses

**Fig. 6:**
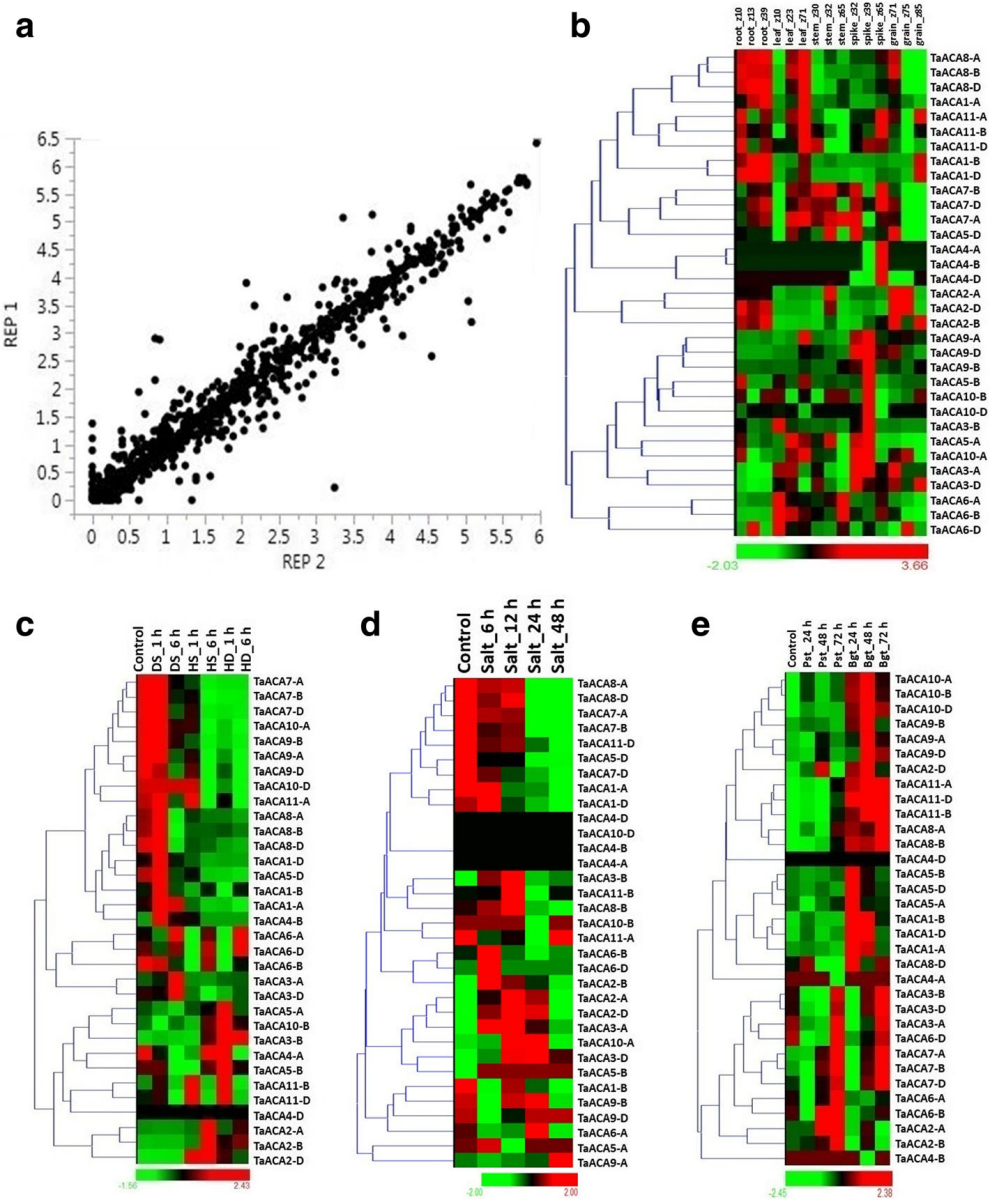
Correlation and expression analyses of *TaACA* genes in different tissue developmental stages, and abiotic and biotic stress conditions. Correlation graph is plotted between biological replicates of expression data derived from tissue developmental stages and abiotic and biotic stresses (**a**). Heat maps showing relative expression profile in three developmental stages of five tissues (root, leaf, stem, spike and grain) (**b**), heat/drought stress (**c**), salt stress (**d**), and under biotic stress (**e**). The developmental stages are shown in Zadoks scale. The differential expression of *TaACA* genes suggested their role during growth and development, and abiotic (heat, drought and salt) and biotic stress conditions

**Fig. 7:**
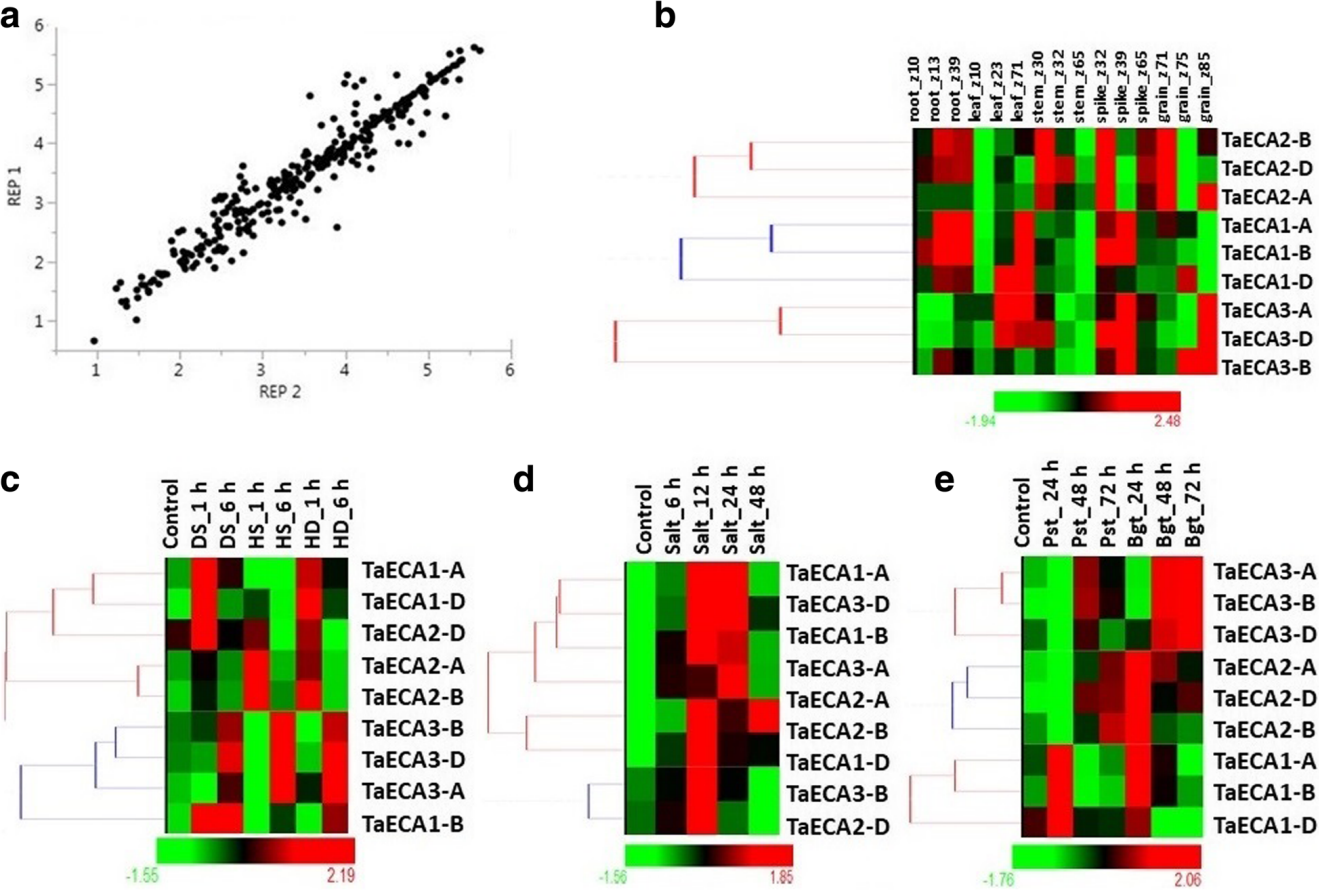
Correlation and expression analysis of *TaECA* genes in different tissue developmental stages, and abiotic and biotic stress conditions. Correlation graph is plotted between biological replicates of expression data derived from tissue developmental stages and abiotic and biotic stresses (**a**). Heat map showing relative expression profile of *TaECAs* in developmental stages (**b**), heat/drought stress (**c**), salt stress (**d**), and under biotic stress (**e**), which indicate their role in growth, development and stress response in *T. aestivum*

The expression data for the replicates of *TaACA* and *TaECA* genes were found consistent with the significant value of correlation coefficient 0.98 and 0.96, respectively (Figs. 6a and 7a).

### Expression analysis in tissue developmental stages

The differential expression of various type II Ca^2+^ATPase genes of *T. aestivum* suggested their role during growth and development. Among *TaACA*s, *TaACA7* and *TaACA8* group genes showed significant expression during almost all the developmental stages of each tissue (Additional file 9: Table S8). Orthologous genes from rice *OsACA10*, *OsACA11* and *OsACA12* also exhibited similar expression [10]. Moreover, *TaACA8-A* and *TaACA8-D* were highly expressed in root, leaf and spike tissues (Fig. 6b). *TaACA3-D* and *TaACA5-D* exhibited high expression during early developmental stages of stem, leaf, spike, and grain. *OsACA6*, found in the proximity of *TaACA3-D* in the phylogenetic tree, also displayed high expression in selected tissue developmental stages [10]. Further, *AtACA7* an ortholog of *TaACA*5-D also showed high expression during early stages of pollen development [12]. Enhanced expression of these genes implied their role during the respective developmental stages, whereas the low expressing genes might be involved in other functions.

In case of *TaECAs*, *TaECA*3*-D*, and each *TaECA1* and *TaECA2* group genes were highly expressed during all tissue developmental stages (Additional file 9: Table S8), which suggested their role throughout the plant development (Fig. 7b). *OsECA3* (LOC_Os05g02940) an ortholog of *TaECA1* group genes is also highly expressed in various developmental stages of tissues [10].

### Expression analysis under heat and drought stress

Since both ACA and ECAs are involved in Ca^2+^ homeostasis, therefore it was expected that their expression would get affected by various abiotic stresses. Earlier studies established the synergistic effect of heat (HS) and drought (DS) stresses [18], therefore the expression analysis was carried out in the presence of HS and DS separately as well as in combination (HD). The genes showing ≥2 fold change in expression value in one or more stages of treatments were described as differentially expressed (Additional file 9: Table S8). A total of 21 *ACA* genes showed differential expression during DS, in which seven genes (*TaACA4-B* and, *TaACA1* and *TaACA3* group genes) were up-regulated, while 14 genes were down-regulated (Fig. 6c). The highly up and down regulated genes were *TaACA3-A* and *TaACA9-D*, respectively. Previously *OsACA6* and *OsACA8* genes of rice were found upregulated in the presence of DS [10], which was phylogenetically related with *TaACA3* and *TaACA9* group genes, respectively. *AtACA4* of Arabidopsis showing downregulation during early stages of DS [10], was found orthologous to *TaACA8*, which was downregulated at DS_6h.

In case of HS, 26 *TaACA* genes were affected, out of which five genes (*TaACA2* group genes and, *TaACA3-B* and *TaACA4-B*) were up-regulated, while the rest were down-regulated (Fig. 6c). The highly up and down regulated genes were *TaACA2-D* (110 fold) and *TaACA9-B* (166 fold), respectively. In HD, out of 24 affected genes, *TaACA3-B* (87 fold) and *TaACA9-B* (208 fold) were highly up and down regulated genes, respectively. *TaACA2* group genes were phylogenetically closer to *OsACA9* (LOC_Os10g28240), which shows high transcript abundance during HS and DS. Furthermore, *TaACA5-D* and *AtACA2*, which are orthologous, showed down regulation during HS [10].

None of the *TaECA* gene expression was significantly affected by DS. However, *TaECA2-A* and *TaECA2-B* were up-regulated after 1 h of treatment of HS and HD (Fig. 7c). The ortholog of *TaECA2* group of genes was *OsECA1*, which also showed somewhat high expression during DS.

We had also analyzed the occurrence of stress specific *cis*-regulatory elements in the differentially expressed genes. Several HS and DS responsive *cis*-regulatory elements such as MYB1AT, MYB2CONSENSUSAT, MYCCONSENSUSAT, ACGTATERD1, DRE, MYCATERD1, MYCATRD22, ABRELATERD1, LTRECORE etc. [36] were detected in the promoter region of differentially expressed genes including *TaACA1*, *TaACA2*, *TaACA3*, *TaACA7*, and *TaACA9*.

### Expression analysis under salt stress

NaCl is known to cause a rapid increase in the level of cytosolic Ca^2+^ thus establishing a link between calcium signalling and salt tolerance in plants [37]. Several studies have established the role of Ca^2+^ATPases in salt stress. An increase in the transcript level of plant endoplasmic reticulum (ER) Ca^2+^ATPases of plants like tomato [31] and tobacco [35] have postulated the involvement of Ca^2+^ pumps in salt stress tolerance. Therefore, we performed the expression profiling of type II Ca^2+^ATPases genes of *T. aestivum* under salt stress.

High FPKM values of these genes in the presence of salt stress suggested their role in salt tolerance (Additional file 9: Table S8). In total, 22 *TaACA* genes were differentially expressed in one or more stages of treatment; out of which eight genes were upregulated while the rest were downregulated (Fig. 6d). *TaACA2* and *TaACA3* group genes were upregulated during all the stages of salt stress examined. The ortholog of *TaACA3* i.e. *OsACA6* is also highly expressed upon salt treatment [10]. Ca^2+^ATPases like *ACA2* and *ACA4* of Arabidopsis have been shown to alleviate salt hypersensitivity in mutant yeast strain [23, 33, 37], which were found evolutionary closer to *TaACA5* and *TaACA7*, and *TaACA4* and *TaACA8*, respectively. Further, *TaACA5*, *TaACA7*, and *TaACA8* group of genes showed differential expression in various stages of salt treatment.

In case of ECAs, only *TaECA*3 group genes showed significant differential expression after 12 h and 24 h of treatment (Fig. 7d). Its ortholog from Arabidopsis i.e. *AtECA*3 also showed an assorted pattern of expression during early and late salt treatments [10].

Among salt-responsive genes, the commonly occurring *cis*-elements were GT1GMSCAM4, ABRE, ACGTATERD, DOFCOREZM, WRKY, MYC, NODCON2GM, MYB and RAV1AAT. Similar elements have also been reported in salt stress-responsive genes in soybean [36].

### Expression analysis under biotic stress

Apart from abiotic stress, plants are also exposed to various kinds of pathogenic infections. The role of Ca^2+^ flux in response to the pathogen’s infection has been established, where an increase in cytoplasmic Ca^2+^ levels is reported [38]. Therefore, we also studied the impact of two fungal pathogens *Blumeria graminis* f. sp. *tritici* (Bgt) and *Puccinia striiformis* f. sp. *tritici* (Pst) of *T. aestivum* to assess the role of P type II Ca^2+^ATPases under biotic stress. Among *ACA* genes, a total of 23 genes were ≥ 2 fold differentially expressed (Additional file 9: Table S8). Out of them, 18 genes were upregulated, and five genes were downregulated under one or more periods of pathogen inoculation (Fig. 6e). The genes that showed highest up and down-regulation were *TaACA1-B* and *TaACA4-A*, respectively. The majority of genes showed dissimilar trends in the treatment of both the pathogens. Moreover, *TaACA1-B, TaACA10-A* and *TaACA10-B* exhibited similar trend in each case of treatment.

Similarly, four *TaECA* genes (*TaECA1-B*, *TaECA2-A*, *TaECA3-A*, and *TaECA3-B*) were upregulated, among which *TaECA3-A* and *TaECA3-B* were upregulated in each hour of Pst and Bgt treatment except 24 h of Pst inoculation (Fig. 7e).

Furthermore, the differentially expressed genes also possessed transcription factors like DOF, WRKY, RAV, MYC, MYB etc. which are involved in plants response during biotic stress conditions [39].

### Interaction analysis and gene ontology (GO) mapping

**Fig. 8:**
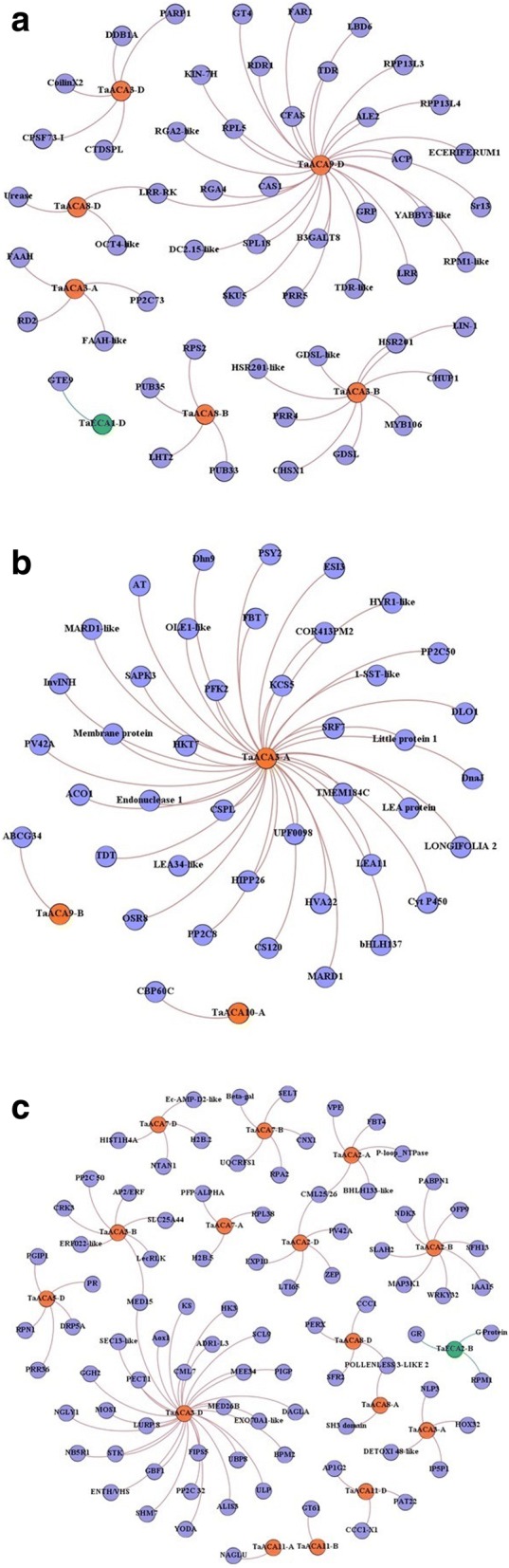
Interaction network of *TaACA* and *TaECA* genes. Interaction networks were plotted using co-expression and Blast2GO data through Gephi 0.9.1 tool for both *TaACA* and *TaECA* genes for tissue developmental stages (**a**); heat/drought stress (**b**) and salt stress (**c**)

The occurrence of various tissue and stress related *cis-*regulatory elements in *type II Ca*^*2+*^*ATPase* genes of *T. aestivum* prompted us to carry out the co-expression analyses in order to establish the potential gene regulatory network. A total of six *TaACA*s and one *TaECA* showed co-expression with 80 genes of *T. aestivum* during tissue developmental stages (Additional file 10: Table S9). *TaACA3-A*, *TaACA3-B* and *TaACA3-D* co-expressed with five, nine and five genes, respectively (Fig. 8a). The co-expressed genes were fatty acid amide hydrolase (FAAH), GDSL esterase/lipase (GDSL), Benzyl alcohol O-benzoyltransferase (HSR201), DNA damage-binding protein 1a (DDB1A), CTD small phosphatase-like protein 2 (CTDSPL) etc. *TaACA8-B* and *TaACA8-D* co-expressed with five and three genes respectively, which included LRR-RKs, organic cation/carnitine transporter (OCT), disease resistance protein RPS2, U-box domain-containing protein 33 and 35 (PUB33 and PUB35) etc. Further, *TaACA9-D* showed co-expression with 52 genes such as beta-1,3-galactosyltransferase 8 isoform X3 (B3GALT8), Leucine Rich Repeat family protein (LRR), probable LRR receptor-like protein kinase (LRR-RKs), receptor-like serine/threonine-protein kinase ALE2 isoform X2 (ALE2), putative disease resistance proteins like RPP13-like protein 3(RPP13L3)/protein 4(RPP13L4), RGA2-like and RGA4 etc. Among *TaECA*s, *TaECA1-D* and gene encoding transcription factor GTE9 were co-expressing (Fig. 8a). Co-expression of *Ca*^*2+*^*ATPases* with the development related genes such as LRR-RKs, FAAH, GDSL, HSR201 etc. suggested their putative interaction network during growth and development. GO mapping of co-expressed genes further revealed their involvement in stress response, and various specific stages of development including anther morphogenesis and tapetum development etc. (Additional file 11: Figure S2A).

During HS, DS and HD three *TaACA* genes showed co-expression with 71 other genes (Fig. 8b). *TaACA9-B* and *TaACA10-A* showed co-expression with ABC transporter G family member (ABCG34) and calmodulin-binding protein (CBP60C), respectively. *TaACA3-A* showing differential expression in the presence of HS and DS co-expressed with the remaining 69 genes, in contrast to the 4 genes during development and salt stress. LEA protein, serine/threonine-protein kinase SAPK3, bHLH, HVA22, invertase inhibitor (InvINH), cold-shock protein CS120, premnaspirodiene oxygenase-like protein (CytP450) etc. were some of the co-expressed genes, which suggested the stress specific role of *TaACA3-A*. GO mapping of co-expressed genes revealed their involvement in carbohydrate metabolism, abscisic acid responsiveness, cation transmembrane transport coupled by ATP hydrolysis, response to water deprivation etc. (Additional file 11: Figure S2B).

During salt stress, six *TaACA*s and one *TaECA* co-expressed with 101 genes (Additional file 10: Table S9). *TaACA3* group genes exhibited co-expression with 48 genes including gamma-glutamyl hydrolase 2 (GGH2), Mov34/MPN/PAD-1 family protein (MEE34), mediator of RNA polymerase II transcription subunit 15-like and 26b (MED15 and MED26B), CDPK-related kinase 3 (CRK3), protein DETOXIFICATION 48-like etc. Further, co-expression of *TaACA2, TaACA7*, *TaACA11* group genes was there with 22, 12 and 5 genes, respectively. Among *TaECA* genes, *TaECA2-B* had co-expression with three genes namely disease resistance protein RPM1, GTP-binding protein (G Protein) and glutathione reductase (GR) (Fig. 8c). Co-expression with salt stress responsive transcripts like cation-chloride cotransporter 1 isoform X1 CCC1, protein DETOXIFICATION 48-like (DETOXI 48-like), MEE34, nucleoside diphosphate kinase 3 (NDK3), polygalacturonase inhibitor 1 (PGIP1), 60S ribosomal protein L38 (RPL38), Disease resistance protein RPM1, Zeaxanthin epoxidase (ZEP) [40] etc. gave supporting evidence for the stress specific role of P-type II Ca^2+^ATPase genes. Annotation and GO mapping hinted towards their role in phosphorylation, DNA-dependent regulation of cellular transcription, protein modification process, cellular response to stimuli like biotic and oxidative stress etc. (Additional file 11: Figure S2C).

## Conclusions

The present study laydowns genome-wide identification of *P-type II Ca*^*2+*^*ATPase* genes in an agronomically important crop plant *T. aestivum*. Among the 42 identified genes, two distinct groups comprising 33 TaACA and 9 TaECA proteins were formed, which were further clustered into 11 and 3 distinct homoeologous groups. An analysis of exon/intron organization revealed three and two distinct groups with close evolutionary relatedness in *TaACA* and *TaECA* genes, respectively. Further, various parameters like length, molecular weight, number of intron/exon, signal peptide, pI and GRAVY score gave additional insight into the characteristics of wheat type II Ca^2+^ ATPase proteins. The TM prediction gave eight to ten TM regions for both the groups of proteins, while for subcellular localization multiple membrane locations were predicted. The motif and domain analysis revealed five major characteristic domains and nine conserved characteristic motifs in Ca^2+^ATPases of *T. aestivum*. The *cis*-regulatory element prediction gave various growth and development, stress, hormonal, and seed-specific and light responsive elements. The differential expression during developmental stages and in the presence of various abiotic and biotic stresses, co-expression with numerous growth and development related and stress responsive genes, and modulated expression during calcium stress suggested vital functions of P-type II Ca^2+^ATPases in *T. aestivum*. However, the *modus-operandi* of each gene needs to be individually validated in future studies. The study thus paves way for the future functional characterization of these genes for crop manipulation and improvement.

## Methods

### Identification, genome-wide distribution, and nomenclature of *type II Ca*^*2+*^*ATPase* genes

We used the protein model sequences of *T. aestivum* (ta_IWGSC_MIPSv2.2) generated by the IWGSC (ftp://ftpmips.helmholtz-muenchen.de/plants/wheat/IWGSC/genePrediction_v2.2) to identify all the type II Ca^2+^ATPase proteins [14, 41] through a local BLASTp search using the reported rice type II Ca^2+^ATPase sequences as a query [10]. All the redundant sequences were removed based on an e-value threshold of 10^− 10^, set for obtaining the final dataset of Ca^2+^ATPase proteins. Subsequently, the Pfam and SMART databases were used to confirm the presence of characteristic P-type II Ca^2+^ATPases domains and motifs. The identified sequences were also searched against the newly reported protein model sequences (TGACv1) of *T. aestivum* (https://plants.ensembl.org/Triticum_aestivum/Info/Index) generated by The Genome Analysis Centre [15] and other available sequences at the NCBI database. The coding sequences were predicted using the FGENESH (http://www.softberry.com/) eukaryotic gene identification program [42].

The type II Ca^2+^ATPase proteins were also identified in *T. urartu* and *Aegilops tauschii* genomes which are the progenitors of A and D sub-genomes of *T. aestivum* [29]. The identified proteins were classified into ACAs and ECAs based on the occurrence of N-terminal autoinhibitory domain and sequence similarity. Nomenclature of the identified genes was carried out following the rules suggested (http://wheat.pw.usda.gov/ggpages/wgc/98/Intro.htm). The identification of homoeologous *TaACA* and *TaECA* genes was performed as described earlier [22, 27]. The chromosomal position was obtained through a BLASTn search against the chromosomal sequences (https://urgi.versailles.inra.fr/blast/, http://plants.ensembl.org/Triticum_aestivum/).

### Sequence alignment and phylogenetic analysis

The sequence alignments were carried out using Multalin, Clustal Omega and Muscle to analyze [43, 44], the conserved regulatory and catalytically significant domains and motifs.

The evolutionary relationship was studied using 110 type II Ca^2+^ATPase protein sequences of *T. aestivum, T. urartu*, *Aegilops tauschii*, rice, and Arabidopsis. The phylogenetic analysis was performed using the full length sequences. An alignment was created using the muscle program [44], which was used for the construction of phylogenetic tree by the maximum likelihood method with 1000 bootstrap replicates using MEGA7.0 [45].

### Structural characterization of genes and proteins

The exon-intron pattern and intron phase distribution were predicted by aligning the coding and genomic sequences using gene structure display server (GSDS 2.0) [46].

To identify the putative TM regions, different tools like TMHMM v2.0, TMMOD, Phobius, SosuiG v.1.1, DAS-Tmfilter and Topcons were employed [47–52]. The conserved domain analysis was done using the SMART [53] and InterProScan [54] servers. The domain architecture map was constructed using IBS server (ibs.biocuckoo.org/online.php). The subcellular localization was predicted employing pSORT, CELLO v.2.5, ngLOC, ProtComp 9.0 (http://www.softberry.com/berry.phtml), MultiLoc2 and BaCelLo servers [55–59] servers. Presence of signal peptide was detected using SignalP 4.1 (http://www.cbs.dtu.dk/services/SignalP/). The ExPasy compute pI/MW/tool was employed for predicting the isoelectric point and molecular weight [60].

### *Cis*-regulatory elements analysis

The genomic sequences of *T. aestivum* were used to retrieve 1500 bp upstream promoter sequences, starting from the translation start site. The occurrence of *cis*-regulatory elements among the retrieved sequences was analyzed using the PlantCARE and PLACE databases [61, 62].

### Expression profiling

The expression value for each gene was calculated in terms of FPKM (fragments per kilobase of transcript per million mapped reads) from high throughput RNA sequencing (RNA seq) data generated in replicates using the Trinity package [63] at p- value 0.001 as described [22, 64]. The expression data were validated through calculation of the Pearson correlation between the replicates using log_2_ (FPKM + 1) values for both *TaACA* and *TaECA* genes, separately.

The tissue-specific expression was analysed employing the data (ERP004714) generated from three different developmental stages of each root, leaf, stem, spike, and grain [17]. The expression pattern was further confirmed at WheatExp server (http://wheat.pw.usda.gov/WheatExp/#).

RNA seq data (PRJNA243835) generated from the leaves of 7-days old seedlings after 24, 48 and 72 h of infestation of fungal pathogens Bgt and. Pst [16] was used to study the impact of biotic stress on expression pattern of *Ca*^*2+*^*ATPase* genes, separately.

The effect of salt stress was studied using the data produced in three biological replicates (PRJNA293629) from root tissues after 6, 12, 24 and 48 h of treatment [19].

Further, the effect of heat (40 °C), drought (20% (m/V) PEG-6000) and their combination was studied using the RNA seq data generated in duplicates (SRP045409) from the leaf samples after one and 6 h of incubation, separately [18].

The genes showing more than two-fold up or down regulation were denoted as differentially expressed. The resulting expression profiles were developed in the form of heat maps using the Hierarchical Clustering Explorer 3.5 (http://www.cs.umd.edu/hcil/hce/). The hierarchical clustering was performed using average linkage obtained by unweighted pair group method with arithmetic mean (UPGMA) and similarity/distance was measured using Euclidean distance at default parameters.

### Interaction analysis

The interaction analyses were performed using co-expression method as described earlier [64]. CoExpress v.1.5 tool [65] was used for identifying the co-expressed genes among the *type II Ca*^*2+*^*ATPases* and other genes of *T. aestivum*. Genes having maximum expression value of ≥5 FPKM and average expression value ≥1 FPKM were used during analyses. Co-expression was computed using Pearson correlation coefficient with a threshold filter of 0.9 and a correlation power 1. Blast2GO tool [66] was employed to determine the annotation and gene ontology (GO) mapping of the co-expressed genes. The interaction network was generated using the Gephi 0.9.1 tool [67].

## Additional files

Additional file 1: **Table S1.** List of protein sequences identified as putative P- type II Ca^2+^ATPase proteins using various databases. The highlighted sequences were full length and showed comparable gene and protein architecture to the reported sequences, and therefore were used during further characterization. (XLSX 15 kb)

Additional file 2: **Table S2.** Clustering of TaACA and TaECA genes from various sub-genomes into homoeologous groups based on high similarity. (XLSX 12 kb)

Additional file 3: **Table S3.** Gene and protein characterization of *T. aestivum* P-type II Ca^2+^ATPases. (XLSX 15 kb)

Additional file 4: **Table S4.** Prediction of number of transmembrane helices in TaACA and TaECA proteins employing different tools. (XLSX 11 kb)

Additional file 5: **Figure S1.** Multiple sequence alignment of *T. aestivum* P-type II Ca^2+^ ATPase proteins. Figure shows full length amino acid sequence alignment of TaACA and TaECA proteins. Transmembrane regions (TM1-TM10) are highlighted by black line on the top; the domains i.e. Cation_ATPase_N, E1-E2 ATPase, Haloacid dehalogenase-like hydrolase (HAD); Cation_ATPase_C are indicated using blue line; CaATP_NAI domain (brown box) and the conserved motifs are also shown. (JPG 4902 kb)

Additional file 6: **Table S5.** Domain architecture analysis of TaACA and TaECA class of proteins. (XLSX 25 kb)

Additional file 7: **Table S6.** Prediction of sub-cellular localization using various tools. (XLSX 11 kb)

Additional file 8: **Table S7.** Prediction of *cis* regulatory elements in *P-type II Ca*^*2+*^*ATPase* genes using PLACE server. (XLSX 233 kb)

Additional file 9: **Table S8.** Expression analyses of *P-type II Ca*^*2+*^*ATPase* genes of *T. aestivum* in various tissue developmental stages and stress conditions. The high expressing genes in tissue developmental stages are highlighted in green boxes. In stress conditions, two fold or more upregulated and downregulated genes are shown in green and yellow colour cells, respectively. (XLSX 25 kb)

Additional file 10: **Table S9.** Annotation and GO mapping of genes co-expressed with *P-type II Ca2 + ATPase* genes of *T. aestivum* during tissue development, heat, drought, and salt stresses. (XLSX 41 kb)

Additional file 11: **Figure S2.** Gene ontology (GO) mapping for the co-expressed genes of *T. aestivum* with *P-type II Ca*^*2+*^*ATPase* genes. GO graph (A) during tissue developmental stages, (B) in the presence of heat, drought and their combination stress and (C) under salt stress. GO mapping was performed using BLAST2GO. (JPG 151 kb)

## Abbreviations

ACA: Auto-inhibited calcium ATPases
Bgt: *Blumeria graminis*
DS: Drought stress
ECA: Endoplasmic reticulum calcium ATPases
HD: Heat-drought combination stress
HS: Heat stress
Pst: *Puccinia striiformis*
