# Supplementary figures and images for: Molecular characterization and differential expression suggested diverse functions of P-type II Ca2+ATPases in Triticum aestivum L
Source: BMC Genomics. 2018 May 23;19:389. doi: 10.1186/s12864-018-4792-9 (PMC5966885; doi:10.1186/s12864-018-4792-9)

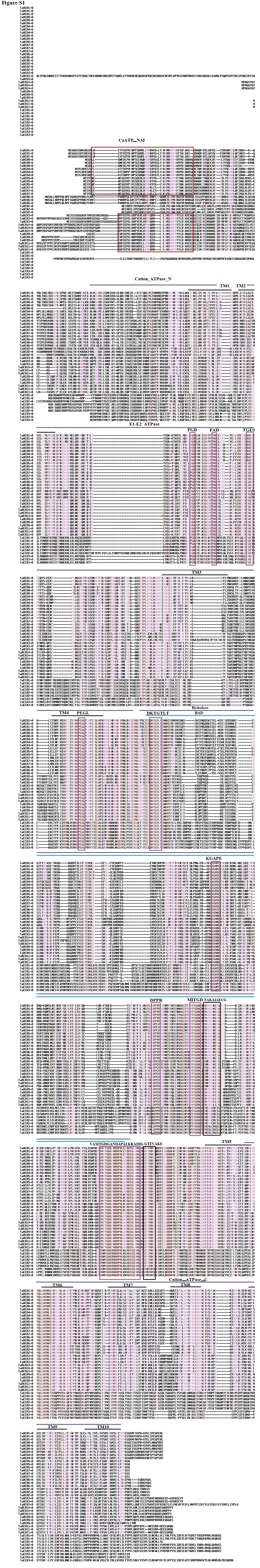

Supplement: Supplementary file 5 — Figure S1. Multiple sequence alignment of T. aestivum P-type II Ca2+ ATPase proteins. Figure shows full length amino acid sequence alignment of TaACA and TaECA proteins. Transmembrane regions (TM1-TM10) are highlighted by black line on the top; the domains i.e. Cation_ATPase_N, E1-E2 ATPase, Haloacid dehalogenase-like hydrolase (HAD); Cation_ATPase_C are indicated using blue line; CaATP_NAI domain (brown box) and the conserved motifs are also shown. (JPG 4902 kb) [file 12864_2018_4792_MOESM5_ESM.jpg]

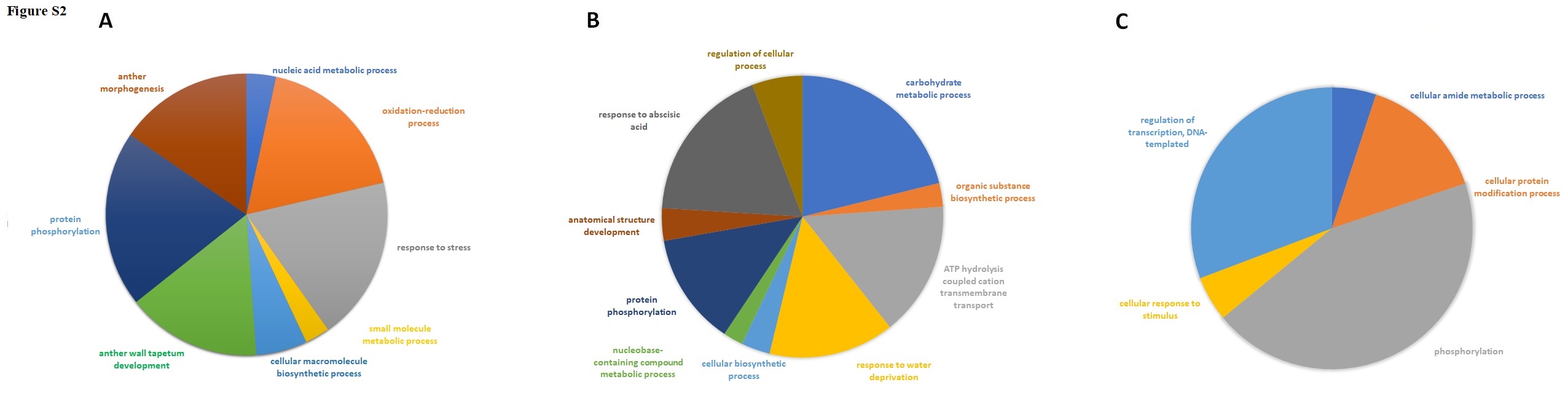

Supplement: Supplementary file 11 — Figure S2. Gene ontology (GO) mapping for the co-expressed genes of T. aestivum with P-type II Ca2+ATPase genes. GO graph (A) during tissue developmental stages, (B) in the presence of heat, drought and their combination stress and (C) under salt stress. GO mapping was performed using BLAST2GO. (JPG 151 kb) [file 12864_2018_4792_MOESM11_ESM.jpg]
